# Successful transfer from insulin to oral sulfonylurea in a 3-year-old girl with a mutation in the *KCNJ11* gene

**DOI:** 10.4103/0256-4947.60526

**Published:** 2010

**Authors:** Maria Al-Mahdi, Angham Al Mutair, Mohammed Al Balwi, Khalid Hussain

**Affiliations:** a From the Pediatric Endocrinology Unit, Pediatric Department, King Abdul-Aziz Medical City, National Guard Hospital, Riyadh, Saudi Arabia; b From the Department of Pediatric, King Fahed National Guard Hospital, King Abdul Aziz Medical City, Riyadh, Saudi Arabia; c From the Department of Pathology and Laboratory Medicine, King Abdulaziz Medical City, Riyadh, Saudi Arabia; d From the Department of Molecular Genetics, Institute of Child Health, University College London, London, United Kingdom

## Abstract

Neonatal diabetes mellitus is considered a rare disease that is diagnosed in the first six months of life, and can be either transient or permanent. Recent advances in molecular genetics have shown that activating mutations in *KCNJ11* (the gene that encodes for the Kir6.2 subunit of the K_ATP_ potassium channel of the pancreatic β-cell) is a common cause of permanent neonatal diabetes mellitus. Patients with mutations in this gene may respond to oral sulfonyureas. We describe a 3-year-old girl with permanent neonatal diabetes mellitus with a mutation in the *KCNJ11* gene (R201H), who was successfully transferred from subcutaneous insulin to oral glibenclamide, with a marked improvement in glycemic control. This is the first successful switch from insulin to oral sulfonylurea in a patient with R201H mutation, in the Arabian Gulf.

Neonatal diabetes mellitus (NDM) is a rare disease with an incidence of 1 in 500 000 newborns. Neonates present with intrauterine growth retardation (IUGR) due to intrauterine insulin deficiency, glucosuria, polyuria, failure to thrive, and ketoacidosis, which usually appear in the first six months of life. Administration of insulin results in a dramatic improvement in the symptoms and growth.^1^ NDM can present as permanent neonatal diabetes (PNDM) or transient neonatal diabetes mellitus (TNDM) that can sometimes be differentiated clinically. Molecular genetic analysis can dramatically differentiate between the two subtypes from the onset of the disease.^2^ The common causes of PNDM are activating mutations in the *KCNJ11* gene, which encodes the Kir6.2 subunit of the K_ATP_ sensitive channel of the pancreatic β-cell.^2^ Mutations in *KCNJ11* cause PNDM in about 53% of the cases.^3^ We report a case with a *de novo* heterozygous mutation in the *KCNJ11* (R201 H) gene that was successfully transformed from subcutaneous insulin to oral sulfonylurea. This mutation results in the inability of the K_ATP_ channel to close, in the presence of increased sensitivity of potassium channel (ATP). It has previously been observed that the introduction of sulfonyurea can close these channels by an ATP-independent mechanism.^4^

## CASE

Our patient was 3 years old when she was diagnosed as having a de novo heterozygous mutation in the *KCNJ11* gene and transfered from subcutaneous insulin to oral glibenclamide. She was born at 40-weeks gestation, with a birth weight of 2 kg, to a healthy mother with no history of gestational diabetes. Her parents were consanguineous with no history of diabetes in the first- or second-degree relatives. At the age of 50 days she was admitted with an acute illness in the form of fever, vomiting, and diarrhea and she was found to have hyperglycemia (blood glucose >20 mmol/L) with no clinical or biochemical evidence of ketoacidosis. As her hyperglycemia was persistent she was started on subcutaneous insulin isophane NPH twice daily (0.3-0.5 units/kg/day). Her initial glycated hemoglobin (HbA1c) was 9% (reference range 4.4-6.4%). Ultrasound of the abdomen showed the presence of pancreatic tissue. A skeletal survey was normal and liver function was normal. She was transferred to us for tertiary care at the age of two years where DNA molecular analysis was done for both parents and patient, after obtaining formal consent. At that time she was clinically well with normal development, and normal physical and neurological assessment. Her HbA1c then was 12% so she was changed to subcutaneous insulin glargine and rapid-acting analogs for better control. Her capillary blood glucose was measured four to six times per day (range of 15-20 mmol/L) with normal diet for her age. There was mild improvement in her HbA1c to 10-11% on changing her insulin regimen.

**Figure 1:**
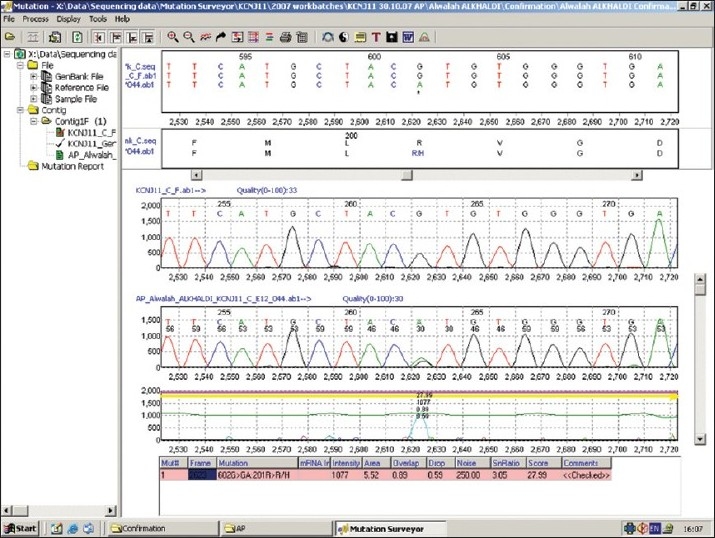
DNA sequence analysis of this patient revealed a de novo heterozygous mutation in *KCNJ11* gene (R201H).

Genomic DNA was extracted from the peripheral leukocytes using standard procedures and the single exon of the *KCNJ11* gene was sequenced as previously described.^5^ Sequencing of the *KCNJ11* gene detected a *de novo* heterozygous mutation in the *KCNJ11* gene (R201H) (Figure 1). At the age of three years the molecular genetic analysis showed that our patient had a *de novo* heterozygous mutation in the *KCNJ11* gene (R201H). The parents were informed and the child was admitted as an in-patient for transfer to oral sulfonylurea glibenclamide. The patient was transferred for a rapid in-patient transfer protocol.^3^ Before starting glibenclamide, the physical examination and neurological assessment were performed, and they were normal for age. Regular capillary blood glucose monitoring was done four to six times a day, the blood was tested for ketones, the HbA1c was checked, and the usual daily dose of insulin was given prior to transfer. On the day glibenclamide was started, an oral glucose tolerance test was performed by giving glucose orally in a dose of 1.75 g/kg. After a sample of fasting blood glucose, insulin level, C peptide was obtained, followed by a postprandial glucose sample. After the oral glucose tolerance test (OGTT) a meal was allowed with rapid acting insulin and the first dose of glibenclamide was given, 0.1 mg/kg/dose, twice daily, in the form of a 5 mg tablet dissolved in water, at a concentration of 5 mg/ml. The following day's long-acting insulin was omitted; rapid acting insulin was continued as necessary, increasing glibenclamide by 0.2 mg/kg/day and continuing capillary blood glucose monitoring. She reached a dose of 0.8 mg/kg/day and insulin was weaned gradually until it was totally withdrawn after eight days of starting oral therapy. The transfer protocol was approved by our institute.

**Figure 2:**
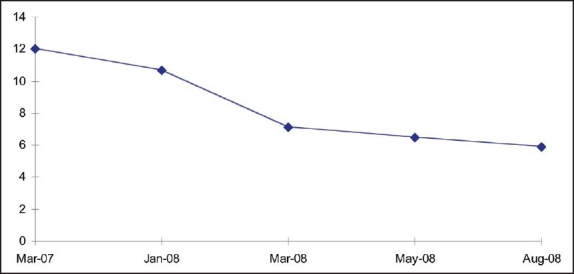
HbA1c before and after switching to glibenclamide.

Our patient was almost three years old when the diagnosis of *de novo* heterozygous mutation in the *KCNJ11* gene (R201 H) was made. At that time she was switched to glibenclamide. Her height was 92.3 cm (twenty-fifth percentile for age), and weight was 14.7 kg (seventy-fifth percentile for age). Before transfer, her HbA1c was 10.7% (reference range 4.4-6.4%), fasting blood glucose was 5.6 mmol/L (reference range 3.3-5.6 mmol/L), the two-hour post-OGTT glucose was 20.1 mmol/L. Her blood glucose was monitored four to six times per day and was in a range of 15-18.6 mmol/L, with insulin of 150 pmol/L, while still on insulin (reference range, 12-150 pmol/L), and C-peptide < 0.5 ng/mL (reference range, 1.1-5.0 ng/mL). She was weaned off insulin 8 days after starting glibenclamide, blood glucose monitoring was continued, with pre-prandial glucose in the range of 3.6-8.2 mmol/L and with midnight monitoring of glucose in the range of 3.6-10.8 mmol/L. Eight weeks later, while still on glibenclamide, with no episodes of hypoglycemia or diarrhea, capillary blood glucose was in the range of 5.5-8.0 mmol/L (decreased from previous recordings), HbA1c decreased from 10.7 to 7.1% (Figure 2), and the insulin level was 19 pmol/L. Twenty-four weeks from starting glibenclamide, she continued to show improvement. Capillary blood glucose monitoring, continued at home, showed that it was almost within the normal range and HbA1c dropped to 5.9%. The patient was compliant with the medications.

## DISCUSSION

It has been shown that mutations in the *KCNJ11* gene are the most common cause of PNDM.^3^ R201H is a mutation in the *KCNJ11* gene that usually presents in isolated diabetes, with no neurological phenotype.^5^ Pearson et al showed the successful switch from insulin to oral sulfonyurea in 90% of patients with *KCNJ11* mutations.^4^

She had regular frequent follow up, eight months from the transfer she needed a reduced dose of glibenclamide (0.5 mg/kg/day), while still maintaining normoglycemic control as observed by Pearson et al.^4^ Other mutations in *KCNJ11* (V59m) have been reported in Arab probands with an associated neurological phenotype,^7^ but to the best of our knowledge, this is the first case of a successful switch from insulin to oral sulfonylurea in the Arabian Gulf, in a patient with R201H mutation. As the mutation in *KCNJ11* is clinically important and there is a proven response to sulfonyurea, it is mandatory to screen patients presenting with diabetes in the first six months, because it will have a major impact on the type of therapy, glycemic control, and quality of life. We have proven that switching from insulin to sulfonyurea was completely successful, without side effects, and the improvement in glycemic control was remarkable with results comparable to other case reports or group studies. However, long-term studies are needed to ensure the long-term safety and effectiveness of sulfonyurea.
