# erratum

**Published:** 2010

In Volume 30, Issue 2, in the following article: Al-Mahdi M, Al Mutair A, Al Balwi M, Hussain K. Successful transfer from insulin to oral sulphonylurea in a 3-year-old girl with a mutation in the *KCNJ11* gene. Ann Saudi Med 2010;30:162-164

An additional affiliation for Maria Al-Madhi should have been Pediatric Endocrinology Unit, Pediatric Department, King Abdul-Aziz Medical City, National Guard Hospital, Riyadh, Saudi Arabia

The corresponding author should be Angham AlMutair, Pediatric Department, Endocrine Division, King Abdul Aziz Medical City, National Guard Hospital, PO Box 22490 Riyadh 11426, Saudi Arabia T: +96612520088 ext. 11630, F: 11641 Mutaira@NGHA.MED.SA

